# A Novel MSPLL-Based Method for Frequency Synthesis in Hydrogen MASER

**DOI:** 10.3390/s26103271

**Published:** 2026-05-21

**Authors:** Dipika Simariya, Sheeba Rani Johnson, Dileep Dharmappa, Suresh Dakkumalla, Prem Ranjan Dubey, Roopa Malali Vasanthakumar, Deva Arul Daniel, Subramanya Ganesh Thirukkodi

**Affiliations:** 1 Avionics Department, Indian Institute of Space Science and Technology (IIST), Trivandrum 695547, India; sheeba@iist.ac.in; 2 ISRO Telemetry Tracking and Command Network (ISTRAC), Navigation Systems Area, Bangalore 560058, India; dileep@istrac.gov.in (D.D.); dsuresh@istrac.gov.in (S.D.); prem@istrac.gov.in (P.R.D.); roopa@istrac.gov.in (R.M.V.); da_daniel@istrac.gov.in (D.A.D.); ganesht@istrac.gov.in (S.G.T.)

**Keywords:** hydrogen maser, phase noise, settling time, loop bandwidth, phase-locked-loop, slave pll, master pll, frequency synthesis, jitter, stability

## Abstract

Frequency synthesis is an important aspect of an atomic clock. It is also imperative that the synthesized frequency exhibits good short term stability or, in other words, exhibits good phase noise. Conventionally single-PLL-system-based approaches have been made for realizing the frequency synthesizers required for hydrogen maser atomic clocks. In this article, a novel approach involving a master–slave-based phase-locked loop (MSPLL) method is presented for frequency synthesis in a hydrogen maser atomic clock. The novelty of this paper lies in the fact that the way two phase-locked loops are coupled to obtain advantage in improving the master oscillator’s stability to match maser physics subsystem stability and at the same time achieving lower jitter by the design. The design involves the usage of a master and a slave phase-locked loop with coupled custom designed direct digital synthesizers for ensuring that the hydrogen maser’s frequency stability is transferred to the master oscillator. The slave PLL (SPLL) generates a low jitter clock for the master PLL (MPLL), thereby guaranteeing reliable tracking of the input reference of 10 MHz, obtained by down-converting the maser physics subsystem frequency of ∼1.4 GHz. A novel mathematical model was derived for the proposed MSPLL design which aids in determination of the settling time of phase, which in turn, leads to the investigation of jitter variance in time domain. A detailed study and analysis of the settling time, phase noise in frequency domain, phase jitter in time domain. and stability performance is presented. The results were validated by the experimental data. The realized frequency synthesizer deduced a settling time of phase that can be adjusted between 689 μs to 811 μs. The synthesized frequency’s phase noise is ≤−114 dBc/Hz at 1 Hz offset, and it was observed that this design induces a very low phase noise to the output signal with respect to the physics subsystem. The achieved short-term stability of the output signal at 1 s is approximately (7.66 × $10^{-12}$) $\tau^{-1/2}$, which is very close to the physics subsystem stability. In terms of stability degradation factor, the proposed MSPLL design exhibits an excellent short-term stability that is one order better than that of the existing methods.

## 1. Introduction

Atomic clocks provide a stable and an accurate frequency output based on the atom’s energy level transitions [1]. Atomic clocks, therefore, find application in satellite navigation, time keeping, etc. The different types of atomic clocks that are commonly used in laboratories and that are commercially available today are cesium clocks, rubidium clocks, and hydrogen masers (active and passive). Out of these, the hydrogen maser provides signals that exhibit excellent short-term stability. In the hydrogen maser, a particular hyperfine level of the hydrogen atom is used to sustain the oscillations inside a microwave cavity, thereby generating a microwave signal at 1420.405751 MHz. The output signal is usually a very feeble signal having a power of 10^−13^ W [2,3]. The hydrogen maser atomic clock consists of two main subsystems namely, the electronic sub-system and the physics subsystem [4]. The electronic subsystem employs a microwave receiver apart from other electronic circuits such as high-voltage source, constant current source, and temperature controller, referred to as "auxiliary systems" [5]. The study presented in this article addresses the research problem of attaining continuous frequency acquisition of physics subsystem’s signal in the microwave receiver while maintaining excellent noise suppression/stability in its final output signal. The microwave receiver’s primary function is to amplify the low power output signal and to down-convert the maser’s output signal (physics subsystem’s signal), which is typically at around 1420 MHz, into a set of lower frequency signals for practical use without degrading the phase noise of the output signal [4,5,6,7,8]. These lower frequencies, referred to as such because the synthesized frequency signals are typically in the range between 1 MHz and 100 MHz, are useful for various precise timing applications, such as timekeeping and satellite navigation. However, the most common output frequency that is used is 10 MHz.

**Figure 1:**
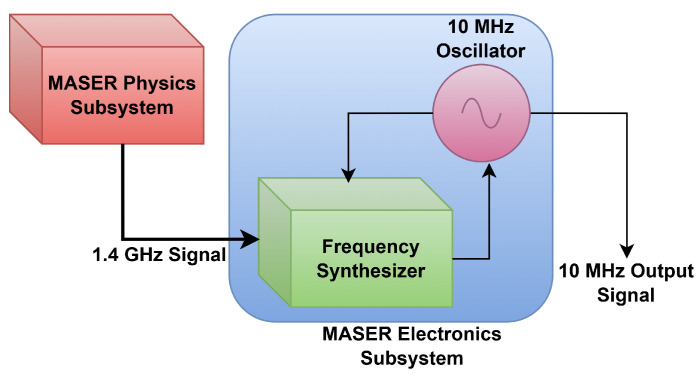
Simplified maser frequency synthesis schematic.

For any hydrogen maser, the stability of its frequency is an important performance metric [9]. The prime emphasis in the design of a microwave receiver is to ensure that the master oscillator’s frequency is phase locked closely to the maser’s output signal [2,10]. The block diagram of the problem being addressed in this article is shown in Figure 1.

As can be seen in Figure 1, the main issue in the generation of a 10 MHz output frequency through the synthesis of maser physics subsystem’s frequency of 1420405751xxx Hz is that the final three numbers in the output frequency are dependent on multiple factors, namely, second-order Doppler shift, wall shift, and applied magnetic field. The small changes in the hydrogen maser frequency output from the physics subsystem necessitates requirement of pll with hundreds of Hz narrow loop-bandwidth. However, due to the instability, inaccuracy, and uncertainties, including the jumps from master oscillator, frequency synthesis is required to have a wide bandwidth of the order of tens of kHz. Having a wider bandwidth will introduce more noise, while having a lower bandwidth cannot ensure continuous lock due to the above-mentioned requirements for hydrogen maser frequency synthesis. In the literature [11,12,13,14,15,16], it is found that to address the tradeoff of loop bandwidth selection, single-pll-based methods are used, but capturing the frequency from the physics subsystem into the microwave receiver and adjusting master the oscillator with respect to this frequency are challenging with the single-phase locked loop. Therefore, a pll system is described in this article which consist of two loop bandwidths. The narrow bandwidth arrests the fluctuations at the Hz level while the wide bandwidth tunes the master oscillator at the kHz level. Thus, the master–slave-based phase-locked loop system can be used to capture physics subsystem’s signal and adapt the master oscillator to that frequency, with the aim of attaining noise and stability characteristics as good those of the physics subsystem’s signal.

The MSPLL-based method for microwave frequency synthesis employs a master–slave configured phase-locked loop along with two direct digital synthesizers in a coupled configuration. In this design, a digitally controllable, stable 10 MHz signal from the primary direct digital synthesizer (pDDS) is generated by locking its signal in the MSPLL system. Unlike earlier synthesizers [17,18,19,20,21,22], this system requires two oscillators, namely, a master oscillator and a slave oscillator. The master oscillator (MO) is a low-noise 10 MHz oscillator’s signal (local oscillator in MPLL), while the slave oscillator (SO) is a low-noise 200 MHz VCXO. The SO is coupled to two direct digital synthesizers. namely, primary DDS (pDDS) and secondary DDS (sDDS). This system requires two PLLs, namely the master PLL (MPLL), which is driven by MO, and the slave PLL (SPLL), which is driven by SO. The proposed method achieves both low phase noise and short-term stability for hydrogen maser.

Furthermore, a clock’s stability is specifically related to the synthesized microwave signal’s settling time and phase noise level [17,18,23,24,25]. Guo et al. [9] presented the design and analysis of the frequency synthesizer for an atomic clock and concluded that the current research is primarily centered on synthesized signal’s low phase noise [19,20,21,22,26,27]. However, there is very little research in the optimization of the settling time. The significance of the settling time is that it is directly related to the jitter variance that occurs in the time domain signal. Mazumdar et al. [28] reported that the reduction in the settling time to a significant value will aid in the minimization of the jitter variance. Therefore, the performance of the designed MSPLL was analyzed by settling time, jitter variance, phase noise, and stability characteristics. To highlight the scope of these contributions, this article is organized into four main sections: Section 2 of this article details the design of the proposed MSPLL method; Section 3 details the mathematical modeling and MATLAB (version R2025b) simulation results for the validation; Section 4 includes experimental data for jitter, phase noise, and stability analysis; and Section 5 presents the conclusion and the scope for future work.

## 2. MSPLL Method for Frequency Synthesis

**Figure 2:**
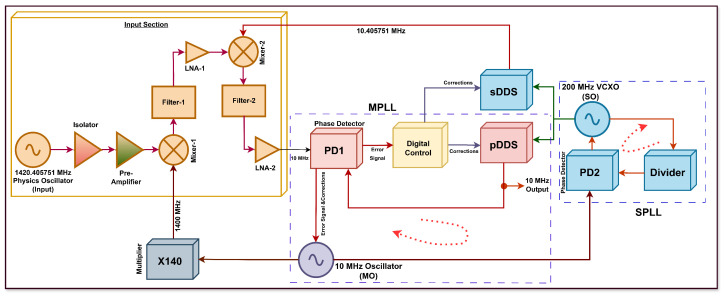
Frequency synthesis chain architecture.

The MSPLL method is presented in Figure 2 and utilizes a master–slave configuration with coupled DDS. This method consists of two oscillators (MO and SO), two PLLs (MPLL and PLL), and two direct digital synthesizers (pDDS and sDDS) along-with filtering, mixing, and multiplication stages. The SPLL maintains low jitter in its clock for the utilization in pDDS and sDDS for stable MPLL signal generation. The proposed method achieves both low phase noise and short-term stability for hydrogen maser.

As shown in Figure 2, a 1420.405751 MHz physics oscillator acts as the input to the frequency synthesis chain. A coaxial type isolator that operates in the frequency range of 800 MHz to 1700 MHz is employed to detect a feeble signal of −100 dBm produced by the physics subsystem by providing an electrical isolation and an electrical safety barrier. It protects the maser cavity (physics subsystem) and receiver front end from reflections, impedance variations, and reverse noise by providing high isolation of at least 50 dB with a low insertion loss of approximately 0.5 dB. Following the isolator, a pre-amplifier, which operates in the frequency band of 1380 MHz to 1520 MHz, is used to amplify the weak signal from the physics subsystem. It ensures minimal degradation of the signal-to-noise ratio prior to the mixing and multiplication stages by offering a 33 dB gain and a noise figure less than 1 dB. This pre-amplification stage decides the noise figure performance of the MSPLL system. After isolation and low-noise amplification, the physics subsystem’s signal is down-converted by using mixing, multiplication, filtering, and amplification, and then the MSPLL-based frequency synthesis is used to produce a usable signal.

The MSPLL-configured frequency synthesis chain comprises two subsystems, namely, the MPLL and SPLL. The MPLL requires two signals, namely, a down-converted reference signal from the physics subsystem and a divided output feedback signal from an oscillator. For the down-conversion of the input signal, firstly, the pre-amplifier’s output is mixed with a 1400 MHz signal using Mixer-1. The 1400 MHz signal is generated using a 140× frequency multiplier which is used to multiply a 10 MHz master oscillator signal (MO). A low-frequency output signal of 20.405751 MHz is obtained from Mixer-1 which is subsequently filtered by Filter-1. Filter-1 offers a pass band of 20 to 21 MHz and a low insertion loss of approximately 2.2 dB. The filtered signal of 20.405751 MHz is amplified by LNA-1, which operates in frequency range of 0.1 MHz to 500 MHz. It raises the signal level to adequately drive the subsequent mixing stage by offering gain of at least 20 dB and noise figure better than 3 dB. After this, the filtered and amplified 20.405751 MHz signal is mixed with a 10.405751 MHz signal generated by sDDS in Mixer-2. The resulting down-converted output of 10 MHz from Mixer-2 is filtered by Filter-2. It operates in 3 to 30 MHz pass band with an insertion loss of approximately 0.49 dB in order to provide sufficient signal level and spectral purity. The output signal of 10 MHz from the Filter-2 is amplified by LNA-2, which has the same characteristics as those of LNA-1, and its output is used as the down-converted signal from physics subsystem to the MPLL. For simplicity, the down-converted physics subsystem signal of 10 MHz is referred to as "input reference" throughout the article. The pDDS and sDDS are operated by SO. This low-jitter stable SO signal is generated from the SPLL by locking it to the master oscillator’s signal, which acts as the reference signal to the phase detector (PD2) of SPLL.

The corrections to the SO with respect to the MO and clean input reference are applied by continuously adjusting their phase and frequency through a negative feedback system in SPLL and MPLL, respectively. In this way, removal of frequency drift is addressed in the output signal from the pDDS through either of the oscillators MO and SO as they are tightly locked to the MPLL and SPLL system, respectively. Hence, when both the MPLL and SPLL are phase-locked, a precise, 10 MHz output signal is available for use at the pDDS. In the phase-locked loop design, the loop bandwidth plays a vital role in achieving desirable noise and jitter characteristics [7,29,30]. The SPLL operates in the loop bandwidth of 253 Hz with the aim of removal of the high frequency noise in the MO, a reference signal to the SPLL. Hence, it will generate a clean, low-jitter signal for the MPLL. Meanwhile, the MPLL’s operational loop bandwidth is approximately 16 kHz in order to acquire the input reference’s signal and adjust the jumps in master oscillator according to it.

### Direct Digital Synthesizer (DDS) Design for MSPLL

The MSPLL model enables determination of the settling time and accordingly the loop bandwidth for the estimation of jitter in the pDDS’s output signal. One of the important requirements in the phase locking of the hydrogen maser’s signal is the generation of a signal of 10.405751 MHz and 10 MHz using the SO clock. The significance of the direct digital synthesis (DDS) is that it provides the output frequency with the high resolution of <1 Hz and offers sub-degree phase tuning by taking into account the frequency error restrictions of the input source. Fixed phase accumulator widths and tuning word resolution are commonly available in default DDS cores, which do not offer the ultra-fine frequency control of the order of 10 pHz or better that is required for the precise synchronization with the hydrogen maser signal. Hence, the direct digital synthesizers pDDS and sDDS coupled to the MSPLL play a crucial role in enabling the high resolution and tunability of the output signal.

In this study, the sDDS and pDDS in the MSPLL system were designed on the Vivado platform with a very high frequency resolution of up to 10 pHz in order to capture subtle changes in the output signal so that excellent spectral purity could be achieved in it. The sDDS is a fractional-based DDS that generates 10.405751 MHz for coupling to the input stage. However, the pDDS is a standard look-up table (LUT)-based DDS which produces an output of 10 MHz. The pDDS and sDDS are made up of a 64-bit phase accumulator (PA) and a 16-bit phase-to-sinusoidal-amplitude mapper (PSAM). The PA bits are truncated to the 16 most-significant bits and mapped to the PSAM. The digital circuit control provides integer and fractional tuning word to pDDS and sDDS, respectively. A dual-channel DAC is employed which uses SO clock to extract two independent DDS outputs by using PSAM bits from both DDSs. Hence, physical signals of 10.405751 MHz from sDDS and 10 MHz from pDDS are generated. By design, the step size is dictated by the Digital Control module shown in Figure 2. The Digital Control module provides a variable step size based on the phase detector output. However, to generate exactly a 10 MHz output, the following control word, in terms of binary bits, is needed: “110011001100110011001100110011001100110011001100110011001101”. The Digital Control module is designed such that the LSB bits of the above-mentioned control word can be varied based on the phase detector output. With the aforesaid control word, the generated output frequency at the output of DDS is calculated as depicted in Equation (1),

(1) $$f_{out}=\frac{M\times f_{clk}}{2^{B}},$$

The $f_{clk}$ = 200 MHz is the clock frequency from SPLL that exactly gives the output 10 MHz while B = 64, which represents number of phase accumulator bits. Since, the Digital Control block can control up to 48 bits, the maximum possible step size can be calculated using *M* = 248, $f_{clk}$ = 200 MHz and *B* = 64 bits as

(2) $$f_{out}=\frac{2^{48}\times 200\times 10^{6}}{2^{64}}\approx 3.05\times 10^{3}\;\mathrm{Hz},$$

Hence, from Equation (2), the maximum possible frequency step size is 3.05 kHz. Due to the large frequency step size, this leads to degraded jitter. However, the frequency steps are applied exclusively by the DDS, and its step size and phase quantization effects have a dominant impact on the overall jitter performance. Therefore, the direct digital synthesis (DDS) design is the major contribution to the design of the MSPLL method for the frequency synthesis in the hydrogen maser. In the next section, the mathematical modeling and MATLAB based simulations are presented for validation of the working of the MSPLL design.

## 3. MSPLL Mathematical Modeling and Simulation

### 3.1. MSPLL Model Analysis

**Figure 3:**
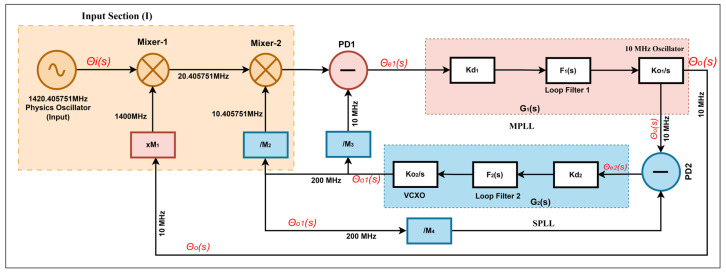
Mathematical model of frequency synthesis chain.

The relationship between the down-converted input reference signal at the input and the PLL’s output can established by defining a transfer function H(s) for the phase of the PLL using control theory [29,31,32], as shown in Figure 3.

As shown in Figure 3, a master–slave configuration is employed for mathematical modeling and analysis of the frequency synthesis chain. In the SPLL, as illustrated in Figure 3, a stable SO clock is used by the pDDS and the sDDS. By using MPLL, a 10 MHz signal is generated from the pDDS that utilizes the SO available in the SPLL. The phase error signal from PD2 in SPLL is defined in terms of $U_{e2}\left(s\right)$. In a complex frequency domain, $U_{e2}\left(s\right)$ can be expressed as

(3) $$U_{e2}\left(s\right)=k_{d2}\Theta_{e2}\left(s\right),$$

where $k_{d2}$ denotes the detector gain of SPLL, and $\Theta_{e2}\left(s\right)$ represents the phase argument of the error signal generated by PD2. Equation (3) presents a linearized model of PD2.

A coupler splits the SO clock into two pathways. The primary path is further subdivided into two additional paths that serve as sample rates to the pDDS and sDDS as well as feedback to PD2. The loop filter’s transfer function in SPLL is defined as $F_{2}$(s), as shown in Figure 3. Therefore, the filter’s error signal can be written as,

(4) $$U_{f2}\left(s\right)=F_{2}\left(s\right)U_{e2}\left(s\right),$$

where $U_{f2}\left(s\right)$ denotes the error signal from the loop filter in the SPLL, which adjusts the SO. In a complex frequency domain, the SO signal corresponding to the signal for control is defined as

(5) $$\Theta_{o1}\left(s\right)=\frac{k_{o2}}{s}U_{f2}\left(s\right),$$

where $k_{o2}$ represents the gain of SO. In Figure 3, the loop gain of SPLL is defined as $G_{2}\left(s\right)$. From Equations (3)–(5), $G_{2}\left(s\right)$ is presented as

(6) $$G_{2}\left(s\right)=\frac{\Theta_{o1}\left(s\right)}{\Theta_{e2}\left(s\right)}=\frac{k_{d2}k_{o2}F_{2}\left(s\right)}{s},$$

The phase detector in SPLL (PD2) generates an error signal $\Theta_{e2}$ which is expressed by Equation (7) in a complex frequency domain as

(7) $$\Theta_{e2}=\Theta_{o}\left(s\right)-\frac{\Theta_{o1}\left(s\right)}{M_{4}},$$

where, $\Theta_{o}$ is the phase argument of the MO signal. Furthermore, $H_{2}\left(s\right)$ in Equation (8) relates the 10 MHz MO to the SO clock in SPLL. Based on Equations (6) and (7), $H_{2}\left(s\right)$ can be expressed as

(8) $$H_{2}\left(s\right)=\frac{\Theta_{o}\left(s\right)}{\Theta_{o1}\left(s\right)}=\frac{k_{d2}k_{o2}M_{4}F_{2}\left(s\right)}{M_{4}s+k_{d2}k_{o2}F_{2}\left(s\right)},$$

Equations (6) and (8) represent the mathematical model of the SPLL. In time domain, it is assumed that SPLL is phase-locked; hence, the SO signal $u_{o1}$ is given by

(9) $$u_{o1}\left(t\right)=a_{1}cos\left(\omega_{2}t+\theta_{o1}\right),$$

where $a_{1}$ denotes the amplitude argument of the signal, and $\theta_{o1}$ and $\omega_{2}$ are the initial phase and radian frequency of the signal, respectively.

The pDDS and sDDS use an SO clock as a sample clock to generate 10.405751 MHz and 10 MHz signals, respectively, as shown in Figure 3. It is assumed that sDDS and pDDS are noiseless divider models. Therefore, the division factors for sDDS and pDDS are $M_{2}$ and $M_{3}$, respectively. Concurrently, the SO clock is used as the divider’s sampling clock; $M_{4}$ is its division factor. The dividers used in the MSPLL design are intended to provide a low-phase-noise feedback signal to the MPLL. The signal derived from the sDDS and the dividers are denoted as $u_{m2}\left(t\right)$, $u_{m3}\left(t\right)$ and $u_{m4}\left(t\right)$, respectively. The calculations for these three signals are as follows:

(10) $$u_{m2}\left(t\right)=a_{m2}cos\left(\frac{1}{M_{2}}\omega_{2}t+\frac{1}{M_{2}}\theta_{o1}\right),$$

(11) $$u_{m3}\left(t\right)=a_{m3}cos\left(\frac{1}{M_{3}}\omega_{2}t+\frac{1}{M_{3}}\theta_{o1}\right),$$

(12) $$u_{m4}\left(t\right)=a_{m4}cos\left(\frac{1}{M_{4}}\omega_{2}t+\frac{1}{M_{4}}\theta_{o1}\right),$$

where $a_{m2}$, $a_{m3}$, and $a_{m4}$ denote corresponding signals’ amplitude.

The signal and center frequency of the master oscillator (MO) in MPLL is represented by $u_{o}\left(t\right)$ and $\omega_{1}$, respectively. Hence, $u_{o}\left(t\right)$, in time domain, is expressed as

(13) $$u_{o}\left(t\right)=a_{o}cos\left(\omega_{1}t+\theta_{o}\right),$$

where $a_{o}$ represents the amplitude, and $\theta_{o}$ is the initial phase of the MO. In MPLL, as illustrated in Figure 3, the 10 MHz signal from the MO is used to drive a multiplier that generates harmonics of 10 MHz. The order of these harmonics is denoted by $M_{1}$ and the $M_{1}$ order of the 10 MHz harmonic as $u_{m1}\left(t\right)$ which can be expressed as

(14) $$u_{m1}\left(t\right)=a_{m1}cos\left(M_{1}\omega_{o}t+M_{1}\theta_{o}\right),$$

where $a_{m1}$ is the amplitude argument of $u_{m1}\left(t\right)$. The signal from input reference $u_{i}\left(t\right)$ is expressed as

(15) $$u_{i}\left(t\right)=a_{i}cos\left(\omega_{i}t+\theta_{i}\right),$$

where $a_{i}$, $\omega_{i}$, and $\theta_{i}$ are the amplitude argument, center frequency, and phase argument of the input reference signal, respectively. The signal $\omega_{i}$ is mixed with a 1400 MHz signal from multiplier in Mixer-1 to generate a 20.405751 MHz signal. The output signal generated by Mixer-1 in Figure 3 is defined as follows $u_{p1}\left(t\right)$:

(16) $$u_{p1}\left(t\right)=\frac{a_{i}a_{m1}}{2}cos\left[\left(\omega_{i}+M_{1}\omega_{o}\right)t+\left(\theta_{i}+M_{1}\theta_{o}\right)\right]+\frac{a_{i}a_{m1}}{2}cos\left[\left(\omega_{i}-M_{1}\omega_{o}\right)t+\left(\theta_{i}-M_{1}\theta_{o}\right)\right],$$

Equation (16) specifies the mixer’s output as $u_{p1}\left(t\right)$, which is divided into two parts: sum and difference. Filter-1 cancels out the high-frequency (sum) signal. The subsequent (difference) part represents a 20.405751 MHz signal, which is amplified by LNA-1 and mixed with a 10.405751 MHz signal from the sDDS in Mixer-2. Its output is filtered by Filter-2, and 10 MHz is captured. The extracted 10 MHz signal from the output of Mixer-2 is amplified by LNA-2 and compared with MO in PD1. From Equations (9)–(16), in the complex frequency domain, the error signal’s phase $\Theta_{e1}$(s) from PD1 is given by

(17) $$\Theta_{e1}\left(s\right)=k_{d1}\left[\Theta_{i}\left(s\right)-\left(M_{1}\Theta_{o}\left(s\right)+\left(\frac{1}{M_{2}}+\frac{1}{M_{3}}\right)\Theta_{o1}\left(s\right)\right)\right],$$

In Figure 3, the transfer function $G_{1}\left(s\right)$ is defined as a loop gain of the MPLL. Thus, the phase argument of the MO ($\Theta_{o}\left(s\right)$) from MPLL, based on Equation (5), is expressed as

(18) $$\Theta_{o}\left(s\right)=\frac{k_{o1}}{s}U_{f1}\left(s\right),$$

(19) $$\Theta_{o}\left(s\right)=\frac{k_{o1}}{s}k_{d1}F_{1}\left(s\right)\Theta_{e1}\left(s\right),$$

In Equation (19), $k_{o1}$ represents the gain of the MO signal, while, $k_{d1}$ and $F_{1}$(*s*) denote the detector gain and loop filter’s transfer function in the MPLL, respectively. Accordingly, the transfer function $H_{1}\left(s\right)$ of the closed loop of MPLL is expressed by

(20) $$H_{1}\left(s\right)=\frac{\Theta_{o}\left(s\right)}{\Theta_{i}\left(s\right)}=\frac{k_{d1}k_{o1}M_{3}F_{1}\left(s\right)}{M_{3}s+k_{d1}k_{o1}F_{1}\left(s\right)H_{2}\left(s\right)},$$

The transfer function H(s) of the closed loop of the MSPLL for the microwave frequency synthesis of the hydrogen maser is shown in Equation (21):

(21) $$H\left(s\right)=\frac{sk_{d1}k_{o1}F_{1}\left(s\right)M_{3}M_{4}+k_{d1}k_{d2}k_{o1}k_{o2}F_{1}\left(s\right)F_{2}\left(s\right)M_{3}}{s^{2}M_{3}M_{4}+sk_{d2}k_{o2}F_{2}\left(s\right)M_{3}+k_{d1}k_{d2}k_{o1}k_{o2}F_{1}\left(s\right)F_{2}\left(s\right)M_{4}},$$

The characteristics of the phase-lock loop transfer function H(s) will determine the overall behavior of the output signal from the locked servo loop, but the phase information is led through an active filter with a spectral response of F(s) and is used to control the phase of the input reference [3]. As shown in Figure 3, loop filter 1 and loop filter 2 individually utilize an active loop filter of the first order, and their mathematical model can be expressed by Equations (22) and (23),

(22) $$F_{1}\left(s\right)=\frac{kp_{1}}{\tau_{1}s+1},$$

(23) $$F_{2}\left(s\right)=\frac{kp_{2}}{\tau_{2}s+1},$$

where $k_{p}$ and τ are the gain and time constant of the active loop filter respectively. The analysis of control loop’s transfer function in the time and frequency domain is presented in Section 3.2.

### 3.2. Simulation Results of MSPLL Design

#### 3.2.1. Evaluation of Transient Response

The phase-locked loop (pll) ensures that the output and the down-converted signal align in phase [29]. However, the PLL takes some time to align its phase due to the instantaneous distortions in the down-converted source’s phase [9]. In this case, the settling time of the phase is very important for the analysis of the PLL-based frequency synthesis [33]. Using Equation (21), simulations of *H*(*s*) were carried out in accordance with the model discussed in Figure 3. The following parameter values were used: $M_{3}$ = 200/10, $M_{4}$ = 20, $k_{d1}$ = 1 V/rad, $k_{d2}$ = 1 V/rad, $k_{o1}$ = 4.49 kHz/V, and $k_{o2}$ = 8.99 MHz/V. The frequency synthesizer has two PLLs; therefore, both the PLLs can have their $k_{p}$ configuration varied during simulation in order to study how they affect the settling time and stability of the system. In terms of hardware, in an active loop filter, changing $k_{p}$ only requires adjusting the gain-setting resistor ratio of the op-amp. However, the loop-filter-time-constant parameter τ in both PLLs are held constant as they require the loop filter hardware to be re-designed. Hence, the parametric analysis of the loop filter time constant τ is omitted.

**Figure 4:**
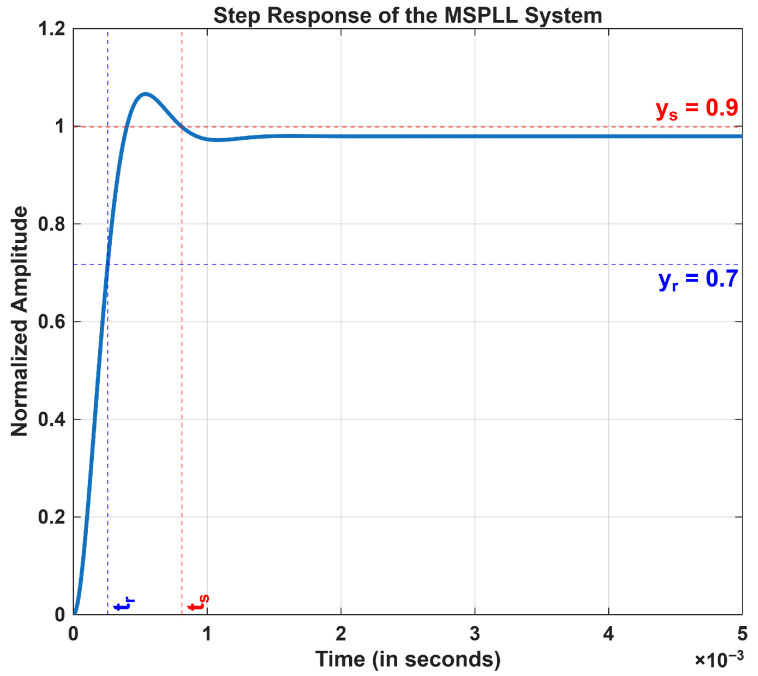
Step analysis with $k_{p1}$ = 2 and $\tau_{1}$ = 0.11 ms.

**Figure 5:**
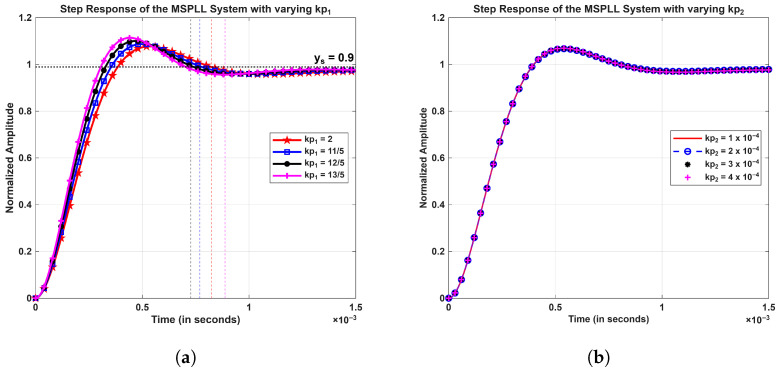
Step response of the phase of output signal (**a**) for $k_{p1}$ varied with fixed $\tau_{1}$ (**b**) for variation of $k_{p2}$ with fixed $\tau_{2}$.

The calculation of the modulated signal’s gain and its usage in the synthesized signal was done by considering $k_{p2}$ and $\tau_{2}$ parameters as fixed at 0.0001 and 1.59 ms, respectively. The resultant parameter values were substituted in Equation (21), and the analysis of the settling time ($t_{s}$) and the rise time ($t_{r}$) in the step responses of the system were investigated and are shown in Figure 4. In the transient response shown in Figure 4, $k_{p1}$ is set to 2, and $\tau_{1}$ is fixed at 0.11 ms, resulting in $t_{s}$≈ 810 μs and $t_{r}$≈ 256 μs. All step responses in Figure 4 and Figure 5 were normalized to unity by applying a scaling factor of 3.3.

Figure 5a shows settling times of the step response of the phase of the output signal for different values of $k_{p1}$ (ranges between 2 and 2.6 in step of 1/5), while $\tau_{1}$ stays at $1.1\times 10^{-3}$ s. The simulated settling times obtained were 810.69 μs, 764.49 μs, 724.63 μs, and 689.432 μs, respectively. The variation of $k_{p2}$ with $\tau_{2}$ fixed at 1.59 ms is shown in Figure 5b, and it shows no major change in the step response of the system. Increasing $k_{p2}$ to tens or hundreds of orders increases the effective bandwidth of the MPLL, which causes an interaction between the MPLL and the SPLL, leading to an ambiguous settling time and reduced stability margins.

**Figure 6:**
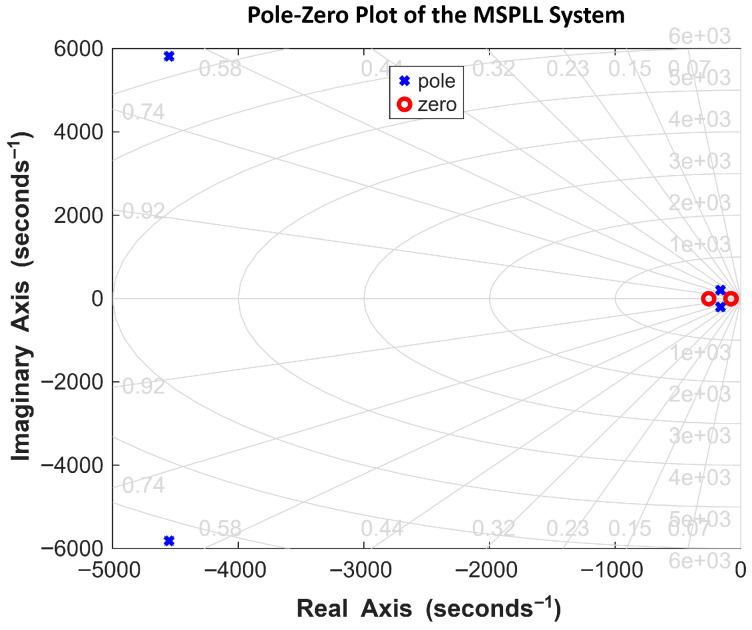
Closed-loop Pole–zero Plot Showing Dominant and Non-Dominant poles and their separation in the complex plane.

There is a tradeoff between the loop filter bandwidth and the suppression level of the fluctuations in the phase of the output signal. It is evident from the simulations shown in Figure 4 and Figure 5 that this tradeoff can be managed well when $k_{p1}$ and $\tau_{1}$ parameters of the loop filter are chosen adequately [9]. Figure 6 shows the closed-loop pole–zero plot of the proposed control loop. All poles are located in the left half of the complex plane, confirming the closed-loop stability. The plot shows two distinct pairs of complex-conjugate poles that exhibit two distinct time scales. The non-dominant pole pair located at approximately s = −4547±j5821 rad/s corresponds to the fast dynamics of MPLL, resulting in a rapid transient that settles within approximately 690 μs to 810 μs. Although these poles dominate the initial transient response, their influence becomes negligible beyond this interval. These fast poles arise from the physical loop filter and oscillator dynamics and are essential for the high-frequency stability, noise suppression, and realizability of the control loop. After this interval, their contribution becomes negligible. The dominant pole pair at s = −98.3±j101.6 rad/s governs the slower dynamics of SPLL and determines the long-term settling behavior of the closed-loop system; however, this slow transient is outside the time scale of interest in the fast-time simulations presented. Section 3.2.2 discusses the steady-state response of the MSPLL system.

#### 3.2.2. Analysis of Steady-State Response

**Figure 7:**
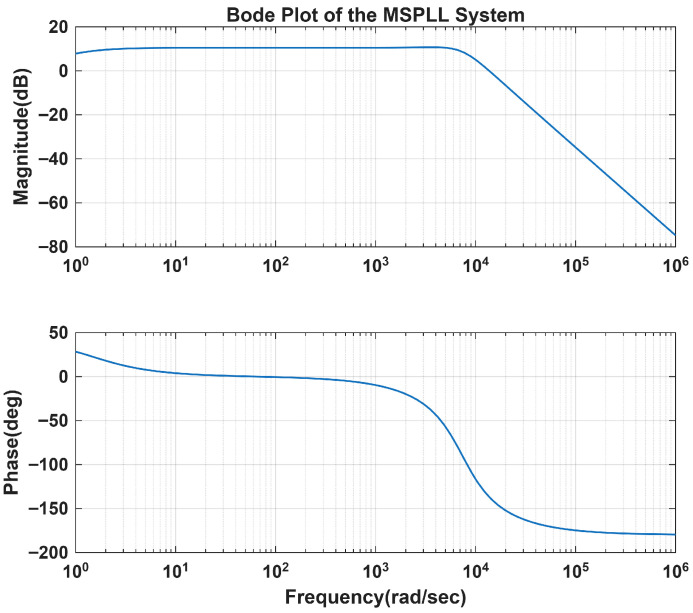
Bode plot of the MSPLL system with $k_{p1}$ = 2 and $\tau_{1}$ = $1.1\times 10^{-4}$.

**Figure 8:**
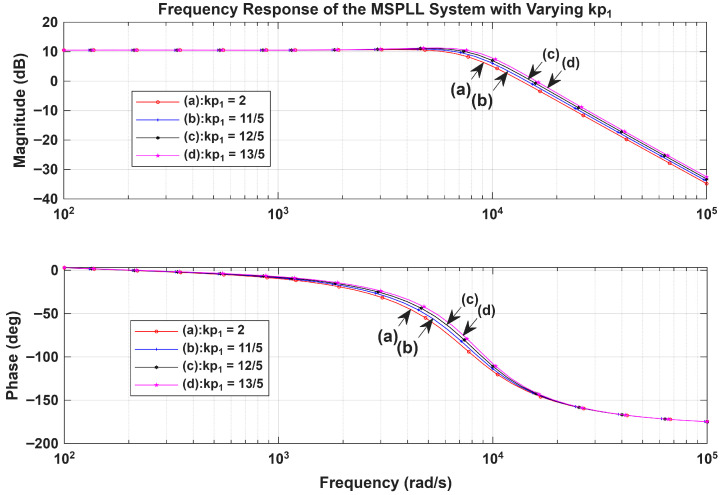
Simulation results showing the Bode plots with variation in $k_{p1}$ and fixed $\tau_{1}$.

The effect of the changes in the MPLL loop filter variables on the transient analysis and on the stability of the system is illustrated in Section 3. The bode plots for assessing the system’s stability and frequency response are presented in Figure 7 and Figure 8.

Figure 8 shows that the system’s stability can be sustained when $\tau_{1}$ and $k_{p1}$ for the loop filters are derived appropriately. In Section 4, the DDS design, its analysis, jitter investigation, and the experimental results for the phase noise and the stability are presented, which enables an end-to-end examination of the proposed method.

## 4. Experimental Results

### 4.1. Experimental Setup

**Figure 9:**
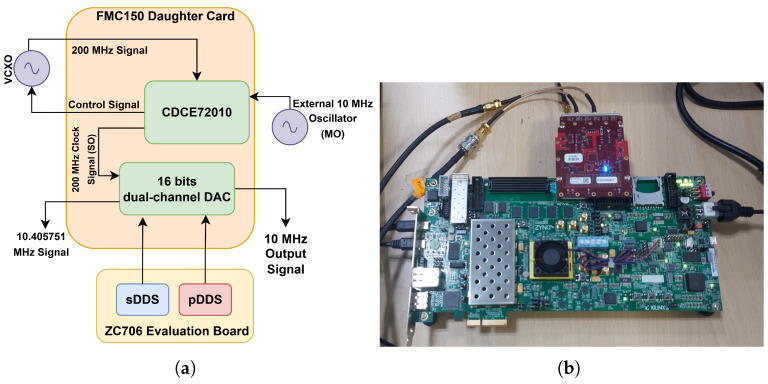
System configuration for signal generation from sDDS and pDDS: (**a**) block diagram of the setup and (**b**) experimental setup.

The generation of the 10.405751 MHz and 10 MHz signals by the sDDS and pDDS, respectively, was carried out by using the system configuration shown in Figure 9.

**Figure 10:**
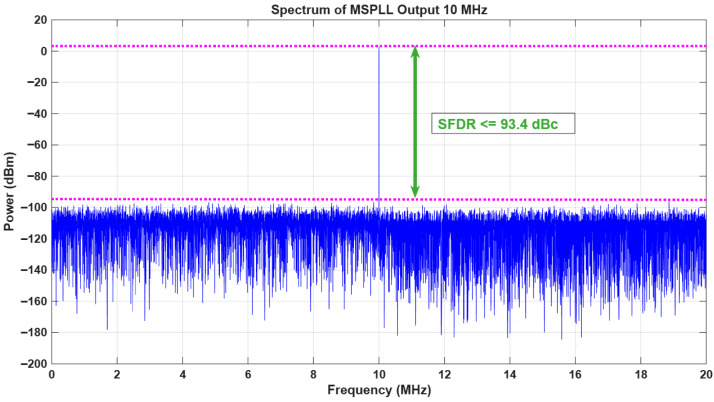
Spectrum of 10 MHz signal from DAC.

Figure 9a provides detailed view of the experimental board setup presented in Figure 9b. It illustrates that the ZC706 evaluation board generate a synthesized frequency of 10.405751 MHz from sDDS and 10 MHz from pDDS. A single die containing the Zynq-7000 (Xilinx Inc., San Jose, CA, USA) XC7Z045-2FFG900C SoC, which combines programmable logic (PL) and an integrated processing system (PS), was achieved by ZC706 evaluation board. The programmable logic (PL) used the Vivado platform for the DDS (direct digital synthesis) to generate the necessary frequency. The VITA 57.1 MPSoC Mezzanine Card (FMC) standard (Abaco Systems Inc., Huntsville, AL, USA) was followed by this evaluation board, which provides low-pin-count (LPC) and high-pin-count (HPC) connection subset versions. The ZC706 board utilized the FMC connector standard on its LPC interface. The FMC connector consisted of two 16-bit D/A and two 14-bit A/D channels, which were timed using a CDCE72010 clock source (with external reference locking) or an additional trigger input provided by an external sample clock for handling the customizable sampling. CDCE72010 clock synchronizer and the jitter cleaner module in the FMC150 daughter card were utilized as the SPLL for the generation of a clean clock by locking the SO to the external 10 MHz reference signal from the master oscillator. The narrow loop bandwidth of 253 Hz was utilized in the FMC interface in order to generate low noise clock from the SO, which was supplied to the pDDS and sDDS as explained in Section 2. Then, a dual-channel digital-to-analog converter (DAC), present in the FMC interface, was used for the generation of the physical 10.405751 MHz and 10 MHz output from the sDDS and pDDS, respectively. The spectrum of the 10 MHz output signal from the pDDS is shown in Figure 10.

#### 4.1.1. Phase Noise Measurement

The theoretical achievable phase noise of the DDS was evaluated by using Equation (24) [34]

(24) $$S_{DDS}\left(F\right)=K_{DDS}^{2}\cdot \left[\frac{10^{k_{2}}}{F^{2}}+\frac{10^{k_{1}}}{F}+10^{k_{0}}\right]+10^{k_{3}}+S_{q}\left(F\right),$$

where

$k_{0}$ = PSD of 1/F^2^ frequency noise,
$k_{1}$ = PSD of 1/F flicker noise,
$k_{2}$ = PSD of thermal noise of DDS DAC,
$k_{3}$ = PSD of the thermal noise of the load,
$K_{DDS}$ (transfer function of DDS) = $\left(\frac{f_{OUT}}{f_{clk}}\right)$,
*F* = frequency offset,
$S_{q}$ (quantization noise of the DDS DAC) = $2^{\left(-2N-0.59\right)}\times \left(\frac{f_{OUT}}{f_{T}^{2}}\right)$,
$f_{T}$ = clock given to the DAC,
and *N* = the number of DAC bits.

(25) $$S_{DDS}\left(10\;\mathrm{Hz}\right)=-113.47\;\;\mathrm{dBc}/\mathrm{Hz},$$

The estimated phase noise in Equation (25) is obtained by considering the generic coefficients for the design of the DDS mentioned in [31,34,35,36,37] as $k_{0}$ = −9, $k_{1}$ = −8.1, $k_{2}$ = −16.6, and $k_{3}$ = −14.1. These values help in approximating the phase noise of the direct digital synthesizer within the range of ± 5 dBc/Hz at the required offset [34].

**Figure 11:**
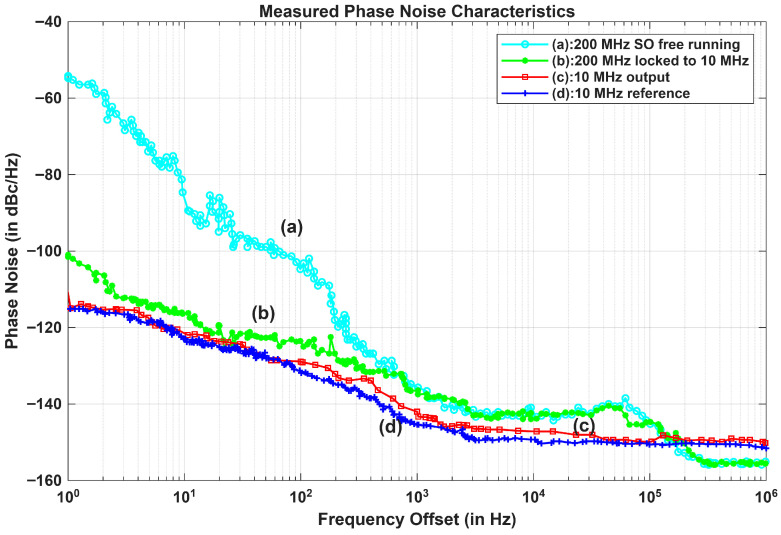
Measured phase noise characteristics.

The measurement of phase noise was performed on the N5511A Phase Noise Test System. The phase noise characteristics of the MSPLL design are shown in Figure 11 and consisted of the obtained phase noise spectra of the 200 MHz SO (free-running and phase-locked), a 10 MHz input reference, and a 10 MHz resultant output signal from pDDS. The measured phase noise values for the input reference were ≤−115 dBc/Hz, ≤−122 dBc/Hz, ≤−130 dBc/Hz, ≤−145 dBc/Hz, and ≤−149 dBc/Hz at the 1 Hz, 10 Hz, 100 Hz, 1 kHz, 10 kHz offset frequencies, respectively. Additionally, the measured phase noise values for 200 MHz SO (free running) were ≤−54 dBc/Hz at 1 Hz offset, ≤−89 dBc/Hz at 10 Hz offset, ≤−103 dBc/Hz at 100 Hz offset, ≤−135 dBc/Hz at 1 kHz offset, and ≤−142 dBc/Hz at 10 kHz offset. When the SPLL locked to the MO (reference to the SPLL), the measured phase noise values obtained from the SO were ≤−101 dBc/Hz at 1 Hz offset, ≤−116 dBc/Hz at 10 Hz offset, ≤−123 dBc/Hz at 100 Hz offset, ≤−136 dBc/Hz at 1 kHz offset, and ≤−142 dBc/Hz at 10 kHz offset. It is evident from Figure 11a,b, that for a carrier frequency of 200 MHz, there is an improvement of nearly 39 dB at an offset frequency of 1 Hz. Hence, a 253 Hz loop bandwidth in SPLL can be effectively employed in order to cope with frequency noise at higher offsets. The comparison of the measured phase noise with that obtained using the earlier designs is shown in Table 1.

The electronics employed in the MSPLL design will cause non-deterministic phase variations by randomly modulating the phase of multiplier’s output signal and adding some thermal noise to the oscillator outputs at different points in the loop [38,39,40]. Therefore, by utilizing the input reference source as a standard, the frequency synthesizer’s phase noise should be assessed according to the real degree of deterioration [9]. As shown in Figure 11c, the phase noise values of the 10 MHz output signal locked to the input reference were ≤−114 dBc/Hz, ≤−120 dBc/Hz, ≤−129 dBc/Hz, ≤−144 dBc/Hz, and ≤−147 dBc/Hz at the 1 Hz, 10 Hz, 100 Hz, 1 kHz, and 10 kHz offset frequencies, respectively. It is concluded that the output signal from pDDS provides optimal phase noise performance close to the phase noise characteristics of the 10 MHz input reference shown in Figure 11c,d through a slight increase in loop bandwidth of MPLL up to 16 kHz and the suppression of the accumulated noise with a narrow bandwidth of 253 Hz in the SPLL.

#### 4.1.2. Jitter Analysis

**Figure 12:**
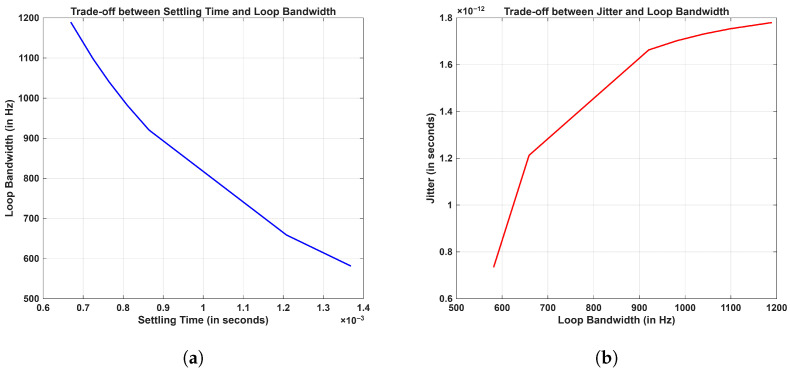
Tradeoffs between (**a**) settling time and loop bandwidth and (**b**) between jitter and loop bandwidth.

The design and the working operation of the MSPLL-based frequency synthesis is presented in Figure 2 and Figure 3. A PLL’s input and output signals, as well as many of its internal signals, are usually binary signals, and the noise that manifests in binary signals is generally described in the form of jitter [41]. The quality of the clock is basically described by the jitter or the phase-noise measurements [42]. Hence, along with the phase-noise analysis of the output signal from pDDS presented in Section 4, the jitter analysis is also described in this section. Kundert [41] proposed that there are two types of jitter. One type of jitter is synchronous, found in blocks such as the PD (phase detector), CP (charge pump), and FD (frequency dividers). It is also known as “phase modulated” (PM) jitter because it appears as a modulation of the output’s phase. The other type of jitter is the accumulation type, which is generally found in crystal type oscillators. The accumulation type of jitter occurs as a modulation of the output frequency, also known as “frequency modulated” (FM) jitter. If the jitter is assumed as either stationary or T-cyclostationary, then the jitter can be measured in terms of edge to edge ($J_{ee}$), period to period ($J_{k}$), or cycle to cycle ($J_{cc}$). If the noise sources are assumed to have a Gaussian distribution (for negligible peak-to-peak jitter), then the relationship between the rms period jitter and the phase noise model can be given by Equation (26) [30,32,41,42,43,44,45],

(26) $$J_{RMS_{period}}=\frac{1}{2\pi f_{c}}\sqrt{\left[\int_{0}^{\infty }2\cdot 10^{\frac{L(f)}{10}}\;df\right]},$$

where $f_{c}$ is the carrier frequency, and *L*(*f*) = phase noise at ‘*f*’ offset in dBc/Hz. Integration bandwidth is crucial in jitter measurement because it defines the frequency range over which timing variations (phase noise) are summed to calculate total jitter, which directly impacts the result. Hence, considering the finite integration bandwidth for theoretical estimation of jitter, Equation (26) is modified as

(27) $$J_{RMS_{period}}{|}_{\left(f_{lb}\;to\;f_{ub}\right)}=\frac{1}{2\pi }\sqrt{\left[2\int_{f_{lb}}^{f_{ub}}\frac{1}{f}\cdot 10^{\frac{L(f)}{10}}\;df\right]},$$

where $f_{lb}$ and $f_{ub}$ are lower and upper bounds of the selected frequency band, respectively. The settling time has an inverse relation with loop bandwidth, and the loop bandwidth has direct a relation with jitter, which are shown in Figure 12a and Figure 12b, respectively. Hence, in order to resolve ambiguity of lower settling with less rms jitter, a loop bandwidth of 16 kHz was selected in the MPLL that is wide enough to settle the loop faster, with the selection of the narrow loop bandwidth of 253 Hz in the SPLL causing less rms jitter accumulation.

**Figure 13:**
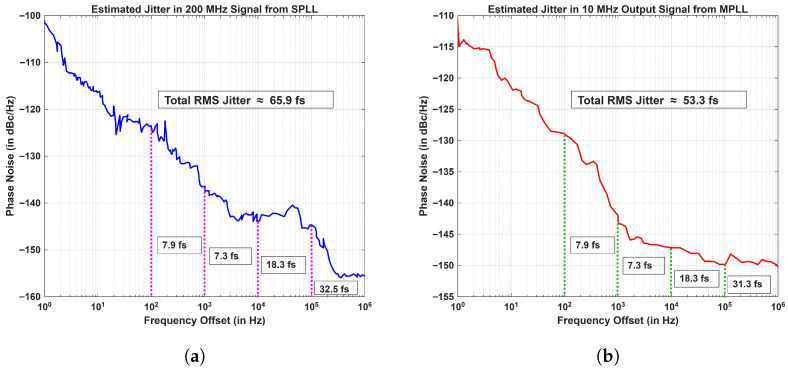
Estimated jitter (**a**) in 200 MHz signal from SPLL and in (**b**) 10 MHz output signal.

The rms jitter estimated from Figure 11 and from Equation (27) in the integration bandwidth of 100 Hz to 1 MHz was 65.91 fs at 200 MHz and 53.26 fs at 10 MHz, as shown in Figure 13a and Figure 13b, respectively. The time-domain jitter measurements were performed using a high-speed oscilloscope R&S RTO2014 (Rohde & Schwarz, Columbia, SC, USA). Given the loop bandwidth parameters in the MSPLL design, the RMS jitter measured for 200 MHz output signal from SPLL was found to be ∼358 fs, and in the case of a 10 MHz signal, the measured value of total RMS jitter was ∼334 fs. The measured values approached the oscilloscope timing noise floor to within approximately 10 to 20%, which indicates optimal measurement performance.

Given the assumption that pDDS is a noiseless divider model incorporated into the VCXO, the noiseless DDS’s output jitter ($J_{DDS}$) originates solely from the jitter at VCXO ($J_{VCXO}$) [41]. For each *N* pulse at the input of the divider, it provides one pulse output such that the output period variation is equivalent to the variance sum in *N* input periods. Mathematically,

(28) $$J_{DDS}=\sqrt{N}\cdot J_{VCXO},$$

By using Equations (26)–(28), rms period jitter for the different synthesized frequencies taken from literature was compared with the rms period jitter computed using the work presented Table 2.

The rate at which the jitter increases becomes more pronounced at the lower output frequencies [46]. In Table 2, Vo is the down-converted physics oscillator’s frequency (in MHz), Vs is the synthesized frequency (in MHz), N is the divide factor, and $J_{i}$ and $J_{o}$ are the calculated rms period jitter (in seconds) for the input reference frequency and synthesized frequency, respectively. Table 2 provides the theoretical effect of the output jitter based on the design of the frequency synthesizer. The MSPLL design proposed in this work can be observed to incorporate less jitter in the produced output frequency compared to that of the existing designs proposed in the literature. This is possible because in the design of the the MSPLL, the value of achievable N is unity. Hence, by design, the MSPLL propagates less jitter to the output synthesized frequency. Therefore this design outperforms the existing methods to provide a low jitter in the output frequency. The low jitter of the output signal directly affects its phase noise and output stability characteristics, which are presented in Section 4.1.1 and Section 4.1.3, respectively. Therefore, the lower the jitter propagation of a given method is, the better will be the output stability and phase noise performance.

#### 4.1.3. Stability Analysis

The short-term frequency stability/Allan deviation can be estimated from the measured single-side-band (SSB) phase-noise spectrum plot [47,48,49,50,51,52,53]. The relation of the phase noise with Allan variance $\sigma_{y}^{2}\left(\tau \right)$ in a generic form for analysis is given by Equation (29) [47,51,54,55],

(29) $$\sigma_{y}^{2}\left(\tau \right)=2\int_{0}^{f_{h}}\left[S_{y}\left(f\right)\cdot \frac{sin^{4}\left(\pi \tau f\right)}{{\left(\pi \tau f\right)}^{2}}df\right],$$

where τ = averaging time (in s), and $S_{y}\left(f\right)$ = SSB fractional frequency noise density = $\frac{f^{2}}{f_{o}^{2}}n\left(f\right)$.

**Figure 14:**
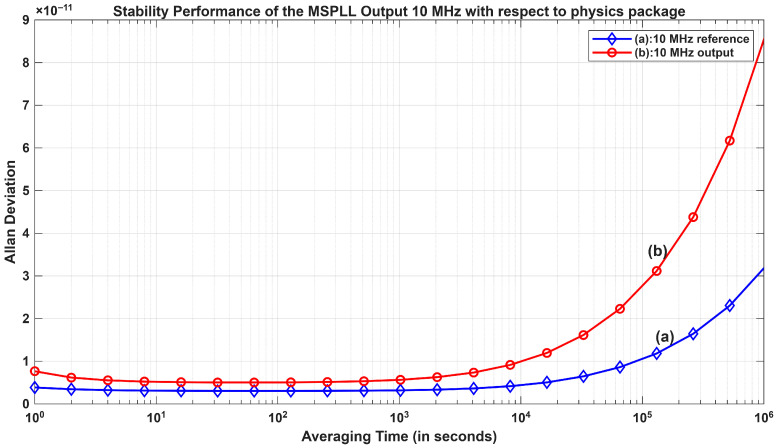
Attained stability of the 10 MHz output signal from pDDS with respect to the 10 MHz physics subsystem (input reference).

For the computation of the Allan variance $\sigma_{y}^{2}\left(\tau \right)$ from Equation (29), the power-law model for the different independent noise processes is applied such that $\sigma_{y}^{2}\left(\tau \right)$ is expressed by,

(30) $$\sigma_{y}^{2}\left(\tau \right)=A_{1}\tau +A_{2}\tau^{0}+A_{3}\tau^{-1}+A_{4}\tau^{-2}+A_{5}\tau^{-2},$$

(31) $$n\left(f\right)=f_{o}^{2}\sum_{\alpha =-2}^{2}\left[h_{\alpha }f^{\beta }\right];\;for\;\;f=\left(0,f_{h}\right],$$

where β = α − 2 is the slope parameter, $f_{o}$ is the carrier or output frequency, n(f) is the SSB phase noise on a linear scale = $10^{\left(L(f)/10\right)}$, L(f) is the SSB phase noise in dBc/Hz, $f_{h}$ is the high cut-off frequency at the phase-noise floor, and $h_{\alpha }$ represents the amplitude coefficient for the different noise processes. The measured stability for the input reference and the phase locked output from the MSPLL is shown in Figure 14 at logarithm (base-10) scale for various averaging times.

As can be seen from Figure 14, the short-term stability of the 10 MHz output from the MSPLL design is as good as that of input reference, aligning perfectly with the aim of the proposed design discussed in Section 1. The comparison of the short-term stability performance of the proposed method and that of the methods described in the literature is shown in Table 3 with respect to the degradation factor (*df*), which is defined as the ratio of the synthesized output stability (*OS*) to the input reference (*IS*) stability. The degradation factor (*df*) is expressed by Equation (32) as

(32) $$\mathrm{df}=\frac{OS}{IS},$$

The comparison of the synthesized output stability ($OS_{2}$) with respect to the input reference ($IS_{2}$) stability utilizing the MSPLL design is denoted by degradation factor $df_{2}$, while $df_{1}$ represents degradation of synthesized output stability ($OS_{1}$) relative to the input reference (($IS_{1}$)) in [56]. From the degradation factors $df_{1}$ and $df_{2}$, it can be surmised that the stability of the MSPLL is one order better than that in the literature [56] at a 1 Hz offset. Additionally, at higher offsets, the degradation factor ($df_{2}$) for the MSPLL design is maintained at <2, which is in contrast to that in [56], in which $df_{1}$ is > 10. Hence, the advantage of the MSPLL design is significant in terms of degradation factor, which was derived for the stability comparison with prior studies. It should also be noted that, for practical experiments, the best available physics subsystem output was considered. Therefore, in terms of degradation factor (*df*), the derived MSPLL approach synthesizes the input reference signal with better short-term stability than do the present techniques.

## 5. Conclusions

A microwave frequency synthesizer was designed using the MSPLL method for use in a hydrogen maser atomic clock. The mathematical model of the MSPLL was derived and the theoretical settling time of phase of the output signal analyzed. The design offers a good settling time and jitter. A precise, low-jitter SO signal is produced by driving the SPLL with a 10 MHz reference from the master oscillator (MO). Then, the MO is adjusted according to the phase of the down-converted physics subsystem signal in the MPLL, and hence, the final output of 10 MHz is derived from the pDDS. The SO signal’s phase noise remains dominant at high offsets, while the physics subsystem’s phase noise is allowed to dominate at low offsets by continuously adjusting the phase and frequency of the MO. The phase noise of the output 10 MHz signal is measured as −114.9 dBc/Hz at 1 Hz offset. Therefore, the MSPLL design achieves very low phase noise at the 10 MHz output signal, which is highly comparable to the physics package. Additionally, an analysis of the jitter from the derived settling time was conducted. The attained short-term stability of the output signal of 10 MHz was 7.65772 × $10^{-12}$, while the physics subsystem short-term stability was 3.85542 × $10^{-12}$, which shows that the proposed MSPLL method for frequency synthesis in the hydrogen maser is able to maintain the physics subsystem’s short-term stability at the 10 MHz output signal. Hence, it is evident that the proposed MSPLL method is able to synthesize the maser frequency output with a superior short-term stability than that of the existing methods in terms of degradation factor (*df*).

## Figures and Tables

**Table 1:** Phase-noise performance comparison.

Offset (Hz)	Guo 2023 [9]		Proposed MSPLL Design	
	200 MHz (Free Running) Phase Noise (dBc/Hz)	200 MHz (Locked to 10 MHz Reference) Phase Noise (dBc/Hz)	200 MHz (Free Running) Phase Noise (dBc/Hz)	200 MHz (Locked to 10 MHz Reference) Phase Noise (dBc/Hz)
1	−56.5	−97.5	−54.2	−101.3
10	−93.2	−110.1	−89.1	−116.4
1000	−145.3	−145.0	−135.7	−136.2
10000	−163.6	−163.8	−142.2	−142.9

**Table 2:** Comparison of frequency synthesization methods for hydrogen maser.

S.No.	Author	Year	Vo (MHz)	Vs (kHz)	N (approx)	Input Jitter $J_{i}$ (s)	Output Jitter $J_{o}$ (s)	Methodology
1.	Levine [14]	1970	5	405.751	12	50.7 femto	178.2 femto	Single-PLL- based methods
2.	Busca [16]	1975	10	11.6	862	17.9 femto	527.1 femto	
3.	Levine [15]	1977	60	405.751	147863	1.2 femto	469.7 femto	
4.	Vanier [13]	1978	10	5.751	1739	17.9 femto	748.6 femto	
5.	Irv Diegel [11]	2013	5	5.751	863	50.7 femto	1.4 pico	
6.	This work	2025	200 and 10 phase-locked to 200	1000	1	17.9 femto	17.9 femto	Master–slave-configured dual PLL with coupled customized DDS

**Table 3:** Short-Term stability performance comparison.

Averaging Time (s)	Wang 2022 [56]		${\mathrm{df}}_{1}$	Proposed MSPLL Design		${\mathrm{df}}_{2}$
	${\mathrm{IS}}_{1}$	${\mathrm{OS}}_{1}$		${\mathrm{IS}}_{2}$	${\mathrm{OS}}_{2}$	
1	2 × 10^−13^	1 × 10^−12^	5	3.85 × 10^−12^	7.65 × 10^−12^	1.9
10	3 × 10^−14^	3.7 × 10^−13^	12.3	3.12 × 10^−12^	5.23 × 10^−12^	1.7
100	7 × 10^−15^	1.16 × 10^−13^	16.5	3.05 × 10^−12^	5.06 × 10^−12^	1.6

## Data Availability

The original contributions presented in this study are included in the article.
